# Integration of High-Resolution Physical and Genetic Map Reveals Differential Recombination Frequency between Chromosomes and the Genome Assembling Quality in Cucumber

**DOI:** 10.1371/journal.pone.0062676

**Published:** 2013-05-06

**Authors:** Qunfeng Lou, Yuhua He, Chunyan Cheng, Zhonghua Zhang, Ji Li, Sanwen Huang, Jinfeng Chen

**Affiliations:** 1 State Key Laboratory of Crop Genetics and Germplasm Enhancement, Nanjing Agricultural University, Nanjing, China; 2 Institute of Vegetables and Flowers, Chinese Academy of Agricultural Sciences, Beijing, China; Nanjing Forestry University, China

## Abstract

Cucumber is an important model crop and the first species sequenced in Cucurbitaceae family. Compared to the fast increasing genetic and genomics resources, the molecular cytogenetic researches in cucumber are still very limited, which results in directly the shortage of relation between plenty of physical sequences or genetic data and chromosome structure. We mapped twenty-three fosmids anchored by SSR markers from LG-3, the longest linkage group, and LG-4, the shortest linkage group on pachytene chromosomes 3 and 4, using uorescence *in situ* hybridization (FISH). Integrated molecular cytogenetic maps of chromosomes 3 and 4 were constructed. Except for three SSR markers located on heterochromatin region, the cytological order of markers was concordant with those on the linkage maps. Distinct structural differences between chromosomes 3 and 4 were revealed by the high resolution pachytene chromosomes. The extreme difference of genetic length between LG-3 and LG-4 was mainly attributed to the difference of overall recombination frequency. The significant differentiation of heterochromatin contents in chromosomes 3 and 4 might have a direct correlation with recombination frequency. Meanwhile, the uneven distribution of recombination frequency along chromosome 4 was observed, and recombination frequency of the long arm was nearly 3.5 times higher than that of the short arm. The severe suppression of recombination was exhibited in centromeric and heterochromatin domains of chromosome 4. Whereas a close correlation between the gene density and recombination frequency was observed in chromosome 4, no significant correlation was observed between them along chromosome 3. The comparison between cytogenetic and sequence maps revealed a large gap on the pericentromeric heterochromatin region of sequence map of chromosome 4. These results showed that integrated molecular cytogenetic maps can provide important information for the study of genetic and genomics in cucumber.

## Introduction

Cucurbitaceae is a moderately family composed of about 130 genera and 900 species [1]. This family contains all gourds crops/cucurbits including economically important crops such as cucumber (*Cucumis sativus* L.), melon (*C. melo* L.), watermelon (*Citrulluis vulgaris* Schrad.), squash and pumpkin (*Cucurbita pepo* L.), and luffa (*Luffa cylindrica* L.). Cucumber has emerged in recent years as a model system for Cucurbitaceae due to its small genomic size of about 367 Mb [2] and some salient biological traits such as sex expression, organelle genetics as well as its economical importance [3]–[5]. Recently, substantial genetic and genomics researches have been conducted in cucumber, including high density genetic maps [6], [7], large insert DNA library such as fosmid [6], and three drafts of genome sequence from Chinese type cucumber [3], American type cucumber [8], and European type cucumber [9]. Despite the current availability of whole genome and genetic resources, the large amount of physical sequences and genetic data cannot be directly related to chromosome structure.

Fluorescence *in situ* hybridization provides a very powerful technique allowing the visual DNA positioning along the chromosome [10]. Mapping the large insert DNA clones, such as BAC, YAC and fosmid anchored by molecular markers onto chromosome enables the generation of cytogenetic map [11]–[14]. Meiotic pachytene chromosomes possess more cytological landmarks and provide superior FISH mapping resolution than somatic metaphase chromosome [15], [16]. Integration of genetic map with physical map based on the high-resolution pachytene chromosome FISH has been reported in crops such as rice, potato, tomato, papaya and maize [17]–[22]. The molecular cytogenetic map of chromosome 2 of cucumber inbred line 9930 was recently constructed based on the fosmid-FISH on pachytene chromosome [23]. Inbred line 9930 is the first cucumber material sequenced with the total length of the assembled genome of 243.5 Mb, about 30% smaller than the genome size estimated [3]. FISH based on high resolution pachytene chromosomes will not only be able to provide a powerful tool to construct the molecular cytogenetic map, but also produce substantial information to polish sequence assembly.

The available high density linkage maps of cucumber showed tremendous difference in length of individual linkage group, ranging from 37.3 cM (LG-4) to 112.7 cM (LG-3) [6]. The knowledge about the existence of difference in chromatin structure and their relationship with genetic recombination frequency are still unknown. In this research, we analyzed the chromatin structures of chromosomes 3 and 4 based on the high-resolution pachytene preparation of cucumber 9930. Twenty-three fosmid clones anchored by SSR markers from the high density SSR map [6] were mapped on pachytene chromosomes to construct the molecular cytogenetic maps of chromosomes 3 and 4. The difference in recombination frequency between chromosomes was compared and their distributions along chromosome were analyzed. The relationships between chromosome structure, gene content and recombination frequency were further investigated. Finally, the cytogenetic map was compared with cucumber draft sequence to evaluate the quality of genome assembly and analyze the distribution of unassembled sequence.

## Materials and Methods

### Plant Materials and Chromosome Preparation

*C. sativus* inbred line 9930 which was used for International Cucumber Genome Project [3] was selected for cytological analysis in this study. The seeds were provided kindly by Dr. Sanwen Huang of Institute of Vegetables and Flowers, Chinese Academy of Agricultural Sciences. The root tips were collected from the geminating seeds for mitotic chromosome preparation. The procedure for mitotic chromosomes was essentially the same as published protocols [24]. For pachytene chromosome preparation, young flower buds were harvested and fixed in 3∶1 Carnoy’s fixative solution for at least 1 day. The anthers at the pachytene stage were digested with enzyme mixtures containing 4% cellulase, 2% pectinase for 1.5 h at 37°C. The digested anthers then were fixed in Carnoy’s fixative solution. The slides with well-spread pachytene chromosomes were obtained by “flame dried” methods [18].

### Chromosome-specific Fosmids and Repeat Sequence Clones

Fosmid clones for physical mapping were obtained from Institute of vegetables and flowers, Chinese Academy of Agricultural Sciences. These clones are sourced from the fosmid library used for cucumber genome sequencing [3]. Chromosome-specific fosmid clones for FISH were selected using SSR markers along linkage groups 3 (LG-3) and 4 (LG-4) of cucumber [6]. Different kinds of satellite DNA sequence [25] including Type I/II, Type III, Type IV, and 45 S rDNA were used for the structural analysis of pachytene chromosomes. Fosmid DNA was isolated using the QIAGEN (Valencia, CA) plasmid midikit.

### Fluorescence *in situ* Hybridization

DNA was labeled with either biotin-16-UTP or digoxigenin-11-dUTP using nick-translation and detected by a fluorescein isothiocyanate-conjugated antibiotin antibody and a rhodamine -conjugated anti-digoxigenin antibody (Roche Diagnostics, Indianapolis, IN), respectively. Chromosomes were counterstained with DAPI in an antifade solution VectorShield (Vector Laboratories, Burlingame, CA), and images were captured using a SenSys CCD camera attached to an Olympus BX51 microscope. The CCD camera was controlled using Applied Spectral Imaging FISH view 5.5 software (Applied Spectral Imaging, Inc, USA) mounted on a computer. The optimal image was performed using Adobe Photoshop (Adobe Systems).

For the physical mapping, we initially used a pair of fosmid probes per slide to orientate their position along chromosome. Thereafter, multiprobe FISH cocktails up to 10 fosmid clone probes were applied to produce consistent measurements. The slides were then used for repeated probing with other fosmids or satellite DNA probes to detect their physical location along the same pachytene chromosome, using the procedure described by Cheng et al. [17].

### Measurement of Pachytene Chromosomes and Construction of Comparative Cytogenetic Maps

The physical location of every fosmid clone was obtained based on the average of measurements at least 8 images. The actual physical distance of fosmid clones along pachytene chromosome was taken according to the method described by Cheng et al. [17] to allow the direct comparison between the physical and genetic maps. The sequence position information in the sequence map was obtained from the published cucumber genome assembly database [3].

### Gene Density Analysis

For the gene density analysis, the numbers of annotated genes per 100 kb along chromosomes 3 and 4 were calculated. We plotted the distribution patterns of gene density along chromosomes. The annotated gene location and number were accessed through Cucumber Genome Database homepage (http://cucumber.genomics.org.cn/page/cucumber/index.jsp).

## Results

### 1. Morphology of Pachytene Chromosomes 3 and 4

**Figure 1:**
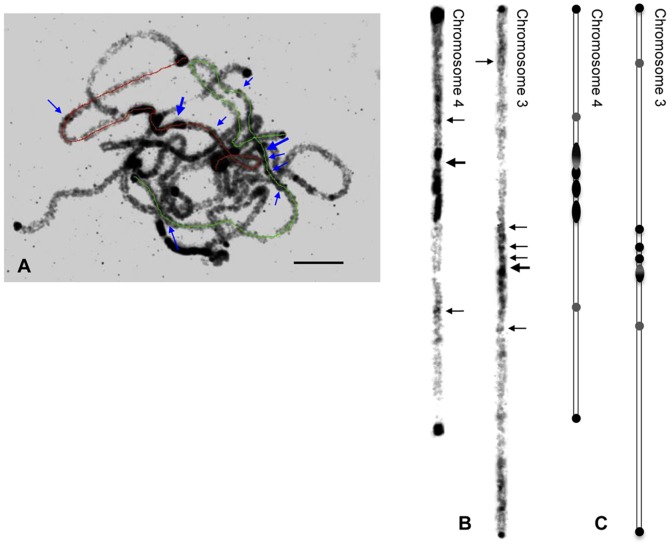
Morphology and heterochromatin distribution on pachytene chromosomes 3 and 4 of cucumber. **A** Pachytene chromosome of cucumber. Chromosome 3 is traced by green line, and chromosome 4 is traced by red line. Identification of individual chromosome was confirmed by FISH mapping of satellites DNA and chromosome-specific fomsids. **B** Computationally straightened chromosomes 3 and 4 from the image shown in (A). Large arrows point to the positions of centromeres, and small arrows point to the knobs in the short and long arm of chromosome 4 and in the region of proximal centromere of chromosome 3. **C** Ideograms of chromosomes 3 and 4. Heterochromatic regions are represented by solid/shaded thickenings. Shaded thickenings indicate the chromosome primary constrictions detected by the probe of centromere specific satellite DNA Type III. These regions were less stained by DAPI compared to the regions of solid thickenings. Bars = 5 µm.

Chromosomes 3 and 4 were identified based on the FISH results of the chromosome-specific fosmid clones and satellite DNAs. The data about chromosome morphological characters is shown in Table 1 and Figure 1. The length of pachytene chromosome 3 varied from ∼42 µm to ∼68 µm, while that of chromosome 4 varied from ∼31 µm to ∼53 µm in different pachytene stages. On average, the length of mid-pachytene chromosomes 3 and 4 of cucumber 9930 was 55.3±3.4 µm and 43.5±3.1 µm, respectively. Chromosome 3 was metacentric and had a long arm of 28.3±2.7 µm and a short arm of 27.1±2.1 µm. The arm ratio of chromosome 4 was ∼1.6∶1, with a long arm of 27.3±2.3 µm and a short arm of 16.1±1.3 µm.

**Table 1:** Length, arm ratio and percentage of heterochromatin, and centromere position of chromosomes 3 and 4.

Chromosome	Totallength (µm)	Longarm (µm)	Short arm (µm)	Arm ratio	Heterochromatin^a^ (%)	Centromereposition^b^ (%)	n
Chr. 3	55.3±3.4	28.3±2.7	27.1±2.1	1∶1	8.9±3.6	49.0±1.9	8
Chr. 4	43.5±3.1	27.3±2.3	16.1±1.3	1.6∶1	24.6±3.2	37.1±1.6	10

a [Total heterochromatin/total chromosome length]×100.

b Centromere position (%) is (S/T)×100, where S = distance of centromere from the end of the short arm, and T = total length of chromosome.

Based on the DAPI staining, chromosomes 3 and 4 showed different heterochromatin and euchromatin distribution patterns, although similar heterochromatin domains were observed at both ends of two chromosomes (Figure 1). Chromosome 3 was mainly euchromatic except for three main knobs adjacent to the centromere in the short arm, and one small knob in both the short arm and the long arm. For chromosome 4, very bright DAPI staining pericentromeric heterochromatin region was observed, and majority of them located on the long arm. And the transition between the heterochromatin and euchromatin regions of chromosome 4 was very sharp. In addition, two interstitial small knobs were also consistently observed on the short arm and the long arm of chromosome 4. Heterochromatin accounted for ∼24.6% of the length of chromosome 4, much higher than that of chromosome 3, ∼8.9% (Figure 1 and Table 1).

### 2. The FISH Patterns of Chromosome-specific Fosmid Clones and Satellite DNAs in Chromosomes 3 and 4

Initially, a set of fifty SSR marker-anchored fosmid clones was chosen for the FISH physical mapping of chromosomes 3 and 4. The fosmid clones were screened based on the FISH signals in somatic metaphase stage. Twenty-three of selected clones that produced bright signals were chosen for the further physical mapping. Among them, 13 clones were from LG-3, while the other 10 were from LG-4 of cucumber (Tables 2 and 3). The relative physical position of individual clone along pachytene chromosome were first investigated based the dual-color FISH. Thereafter, multi-probe FISH cocktails were hybridized to the same pachytene chromosome to obtain more accurate measurements.

**Table 2:** Genetic and physical position of SSR marker-anchored cucumber chromosome 3-specific fosmid clones and satellite DNAs.

CloneCode	Clone (Fosmid) ID	Sequence position(bp)	SSR marker	Chromosome arm	cM	FL^a^	n
Ty IV	Type IV	–	–	Long arm	–	0	9
Ty I/II	Type I/II	–	–	Long arm	–	0	9
3–1	rgcfbe0_0472_B12.ab1	1121698	SSR03049	Long arm	0	6.0±1.1	10
3–2	gcfbe0_0207_F03.ab1	10059164	SSR22514	Long arm	31.0	29.0±1.9	12
3–3	gcfbd0_0441_G08.ab1	12493789	SSR13274	Long arm	40.7	37.0±1.8	11
3–4	gcfbd0_0008_A02.ab1	16190322	SSR02244	Long arm	14.7	46.3±2.0	13
3–5	gcfbd0_0554_G09.ab1	16484183	CSWGATT0	Long arm	49.4	47.1±1.3	9
3–6	gcfbd0_0392_E05.ab1	19248575	SSR14526	Long arm	44.4	52.5±1.0	8
45S	45S rDNA	–	–	Long arm	–	55.3±2.2	10
Ty III	Type III	–	–	Centromere	–	56.7±1.6	9
3–7	gcfbd0_0286D12.ab1	21468111	SSR21326	Short arm	–	58.3±1.3	8
3–8	gcfbd0_0643G06.ab1	23264296	SSR12032	Short arm	59.0	61.7±1.6	8
3–9	gcfbd0_0073H09.ab1	25165760	SSR04632	Short arm	62.4	65.0±1.8	9
3–10	gcfbe0_0072_E12.ab1	30295325	SSR05572	Short arm	84.7	83.1±1.6	11
3–11	gcfbd0_0515_B12.ab1	31717595	SSR03777	Short arm	90.0	86.4±2.0	6
3–12	gcfbe0_0104_H02.ab1	32147183	SSR21454	Short arm	92.4	86.8±1.4	8
3–13	gcfbe0_0001_D11.ab1	38050645	SSR23517	Short arm	105.1	106.9±2.4	10
Ty I/II	Type I/II	–	–	Shortarm	–	112.7±1.8	9
Ty IV	Type IV	–	–	Shortarm	–	112.7±1.8	9

a Fraction length (FL) = (S/T)×112.7, where S is the distance (micrometers) from the FISH site to the end of the short arm, T is the total length of the chromosome (micrometers), and 112.7 is the length (in centimorgans) of the linkage map of chromosome 3.

**Table 3:** Genetic and physical position of SSR marker-anchored cucumber chromosome-4 specific fosmid clones and satellite DNAs.

CloneCode	Clone (Fosmid) ID	Sequence position(bp)	SSR marker	Chromosome arm	cM	FL^a^	n
Ty IV	Type IV	–	–	Long arm	–	0	10
Ty I/II	Type I/II	–	–	Long arm	–	0	10
4–1	gcfbe0_0315F11	351	–	Long arm	–	0.7±0.6	8
4–2	gcfbd0_0280_E09.ab1	1301733	SSR03598	Long arm	0	1.9±0.3	8
4–3	gcfbe0_0162_A08.ab1	7374241	SSR01601	Long arm	4.4	9.5±1.4	9
4–4	gcfbd0_0476H06	10490315	–	Long arm	–	12.1±1.8	7
Ty III	Type III	–	–	Centromere	–	13.5±1.6	10
45S rDNA	45S rDNA	–	–	Short arm	–	14.7±0.8	10
4–5	gcfbe0_0243_E12.ab1	11480614	SSR23826	Short arm	7.5	20.0±2.0	9
4–6	gcfba0_0090_G08.ab1	11633420	SSR14617	Short arm	9.3	20.7±0.8	10
4–7	gcfbe0_0115_C08.ab1	12425172	SSR19565	Short arm	16.2	22.4±0.6	10
4–8	gcfbe0_0098_F07.ab1	19347769	–	Short arm	–	30.2±1.4	9
4–9	gcfbd0_0077_A03.ab1	20434117	–	Short arm	–	32.2±1.3	8
4–10	gcfbd0_0802_E07.ab1	23135337	SSR30478	Short arm	35.1	35.9±1.1	6
Ty I/II	Type I/II	–	–	Short arm	–	37.3±1.9	10
Ty IV	Type IV	–	–	Short arm	–	37.3±1.9	10

a Fraction length (FL) = (S/T)×37.3, where S is the distance (micrometers) from the FISH site to the end of the short arm, T is the total length of the chromosome (micrometers), and 37.3 is the length (in centimorgans) of the linkage map of chromosome 4.

**Figure 2:**
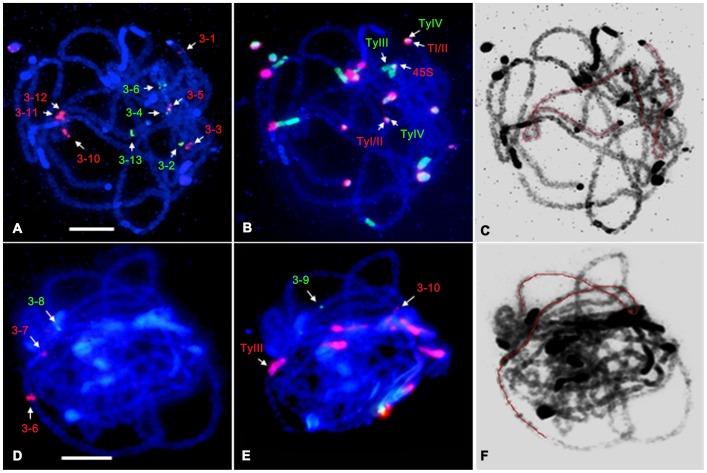
FISH mapping of cucumber chromosome 3-specific fosmid clones and satellite DNAs on the pachytene chromosome 3. **A** Fish mapping of 10 fosmid clones on the pachytene chromosome 3 of cucumber. **B** Fish mapping of 45S rDNA (red), Type I/II (red), Type III (green), and Type IV (green) clones on the same slide as A using reprobing method. **C** The DAPI-stained chromosomal image was converted as a black–white image to enhance the visualization of chromosome structure. **D** Fish mapping of 3 fosmid clones on the pachytene chromosome 3 of cucumber. **E** Fish mapping of Type III (red) and two fosmid clones on the same slide as D using reprobing method. **F** The DAPI-stained chromosomal image was converted as a black–white image to enhance the visualization of chromosome structure. The pachytene chromosome 3 was orientated with red line. Bars = 5 µm.

**Figure 3:**
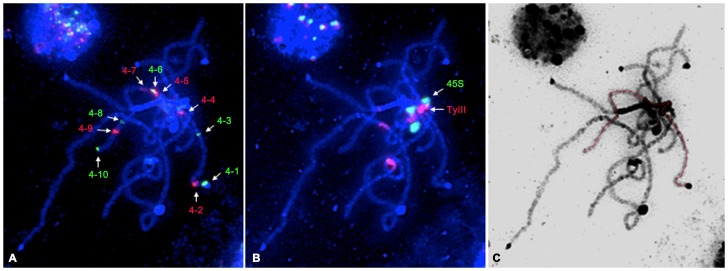
Fish mapping of cucumber chromosome 4-specific fosmid clones and satellite DNAs on the pachytene chromosome 4. **A** Fish mapping of 10 fosmid clones on the pachytene chromosome 4 of cucumber. **B** Fish mapping of 45S rDNA (green) and Type III (red) clones on the same slide using reprobing method. **C** The DAPI-stained chromosomal image was converted as a black–white image to enhance the visualization of chromosome structure, and the pachytene chromosome 4 was orientated with red line. Bars = 5 µm.

We used several sets of cocktail probes by overlapping some probes to produce comparable measurements in different chromosome preparations (Figures 2A and 2D). Meanwhile, the chromosome preparations were then reused with other fosmids or satellite DNA probes to produce the data for physical distance of all clones and to investigate the structures of pachytene chromosome 3 (Figures 2B and 2E) and chromosome 4 (Figures 3A and 3B).

For chromosome 3, six clones from 3–1 to 3–6 were positioned on short arm and seven clones from 3–7 to 3–13 were positioned on long arm (Figures 2A, 2D and 2E). 45S rDNA produced signal in the heterochromatin region of short arm, adjacent to Type III which was located in centromeric regions. The other two kinds of satellite DNAs, Type I/II and Type IV were located in the telomeric heterochromatin regions of pachytene chromosomes. FISH signals showed Type I/II and Type IV partially overlapped and the Type IV was distal compared to Type I/II (Figure 2B).

For chromosome 4, four fosmid clones from 4–1 to 4–4 were positioned on short arm and other 6 clones from 4–5 to 4–10 were located on long arm (Figure 3A). Unlike chromosome 3, 45S rDNA was positioned in the heterochromatin region of long arm, adjacent to Type III, centromere region (Figure 3B). Clones 3–4 and 3–5, clones 3–11 and 3–12, clone 4–5 and 4–6 were positioned very closely, but their order could be distinguished based the signals in early pachytene stage chromosome (unpresented data). To show the structure clearly, the DAPI-stained pachytene chromosomes 3 and 4 were converted to a black-white image and the trends were highlighted with red lines (Figures 2C and 3C).

### 3. Correlation between Genetic and Physical Distance on Cucumber Chromosomes 3 and 4

**Figure 4:**
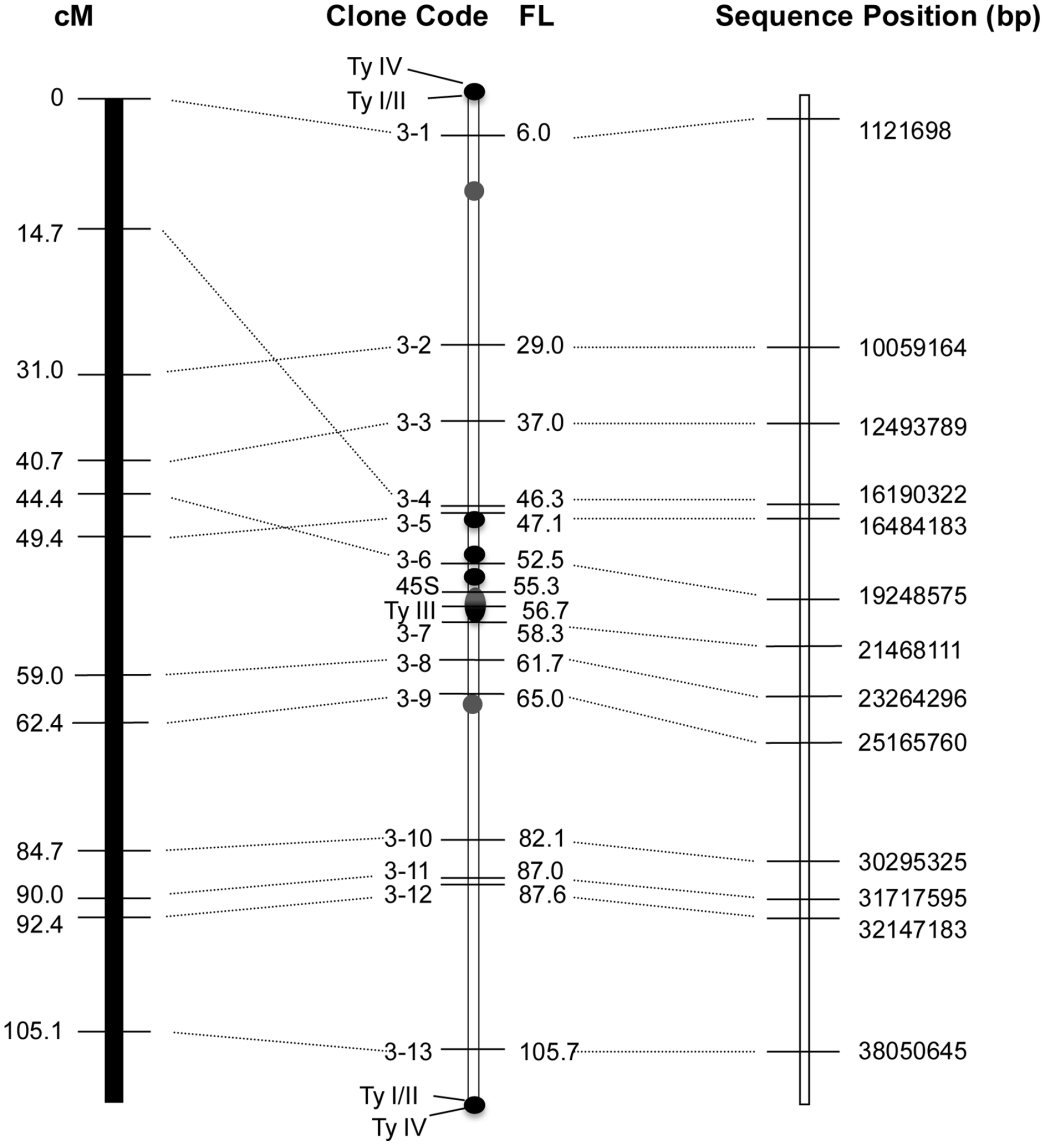
Integration of genetic linkage map of cucumber chromosome 3 with cucumber pachytene chromosome 3. The genetic linkage map of cucumber chromosome 3 on the left lane is according to Ren et al. (2009). The sequence map of cucumber chromosome 3 on the right lane is according to Huang et al. (2009).

**Figure 5:**
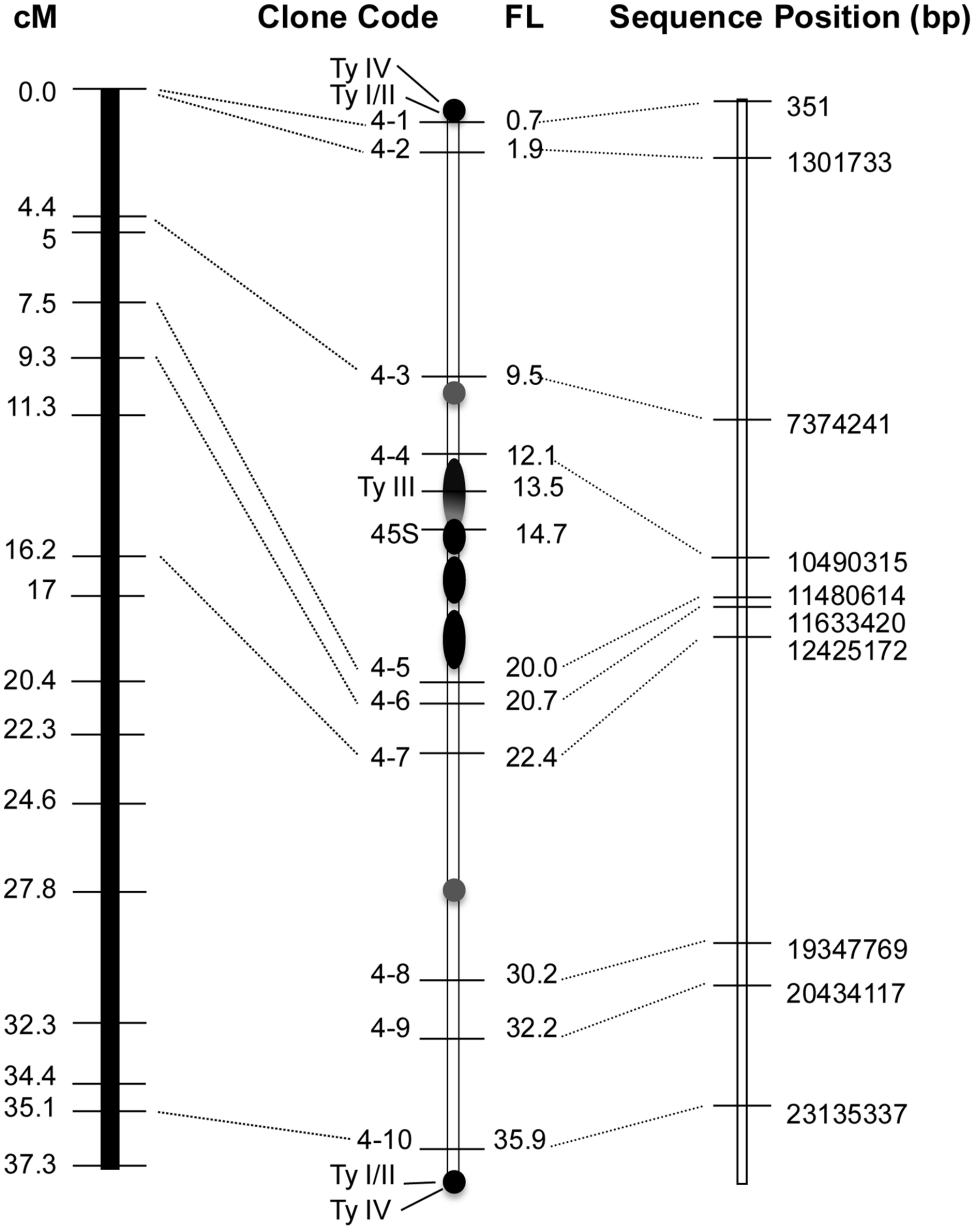
Integration of genetic linkage map of cucumber chromosome 4 with cucumber pachytene chromosome 4. The genetic linkage map of cucumber chromosome 4 on the left lane is according to Ren et al. (2009). The sequence map of cucumber chromosome 4 on the right lane is according to Huang et al. (2009).

The relative cytological position of individual fosmid was calculated and the results were listed in Tables 2 and 3. The total genetic length of LG-3 and LG-4 of cucumber are 37.3 cM and 112.7 cM [6]. And the selected SSR markers were distributed at an average distance of ∼4 cM and ∼10 cM along the LG-3 and LG-4, respectively. The signals of fosmid clones and satellite DNAs were measured on a minimum of 8 pachytene chromosome spreads. Clones 3–1 and 3–13 anchored by SSR markers, SSR03049 and SSR23517 from the both ends of LG-3 respectively produced signals close to the end of pachytene chromosome 3. About 91.2% of the length of pachytene chromosome 3 was covered by these two SSR markers-anchoring fosmids (Figures 1 and 4). Similarly, over 97% of the length of chromosome 4 was covered by clone 4–1 (located at 0.6 FL) and clone 4–10 (located at 36.2 FL) (Figure 5). These results indicated the high coverage of the genetic maps of chromosome 3 and chromosome 4.

The physical orientation of fosmid clones in chromosome 3 was concordant with that of SSR markers along the LG-3 except for three markers, SSR13274, SSR02244 and CSWGATT0 (Table 2 and Figure 4). Physical mapping results showed the marker SSR02244 (14.7 cM) was positioned between the markers SSR13274 (40.7 cM) and CSWGATT0 (49.4 cM). This result indicated that SSR 02244 is likely mapped wrongly on the genetic map. The positions of markers CSWGATT0 (fosmid clone 3–5) and SSR14526 (fosmid clone 3–6) were reversed on the cytological map compared with their locations on the genetic map (Figure 4). The order of all fosmid clones along chromosome 4 was concordant with the order of the SSR markers along the LG-4 (Table 3 and Figure 5).

The pachytene FISH results also could differentiate the tightly linked markers, especially for those located in heterochromatin regions. For example, SSR markers anchoring fosmid clones 4–1 and 4–2 were both mapped to 0.0 cM on the genetic map (Table 2). In physical map, however, 4–1 and 4–2 were located on short arm at 0.6 FL and 2.0 FL, respectively.

### 4. The Disparity of Recombination Frequency Along Chromosome and between Chromosomes 3 and 4

Overall, the recombination frequency along chromosome 3 seemed to be not significantly different (Figure 4). In contrast, the recombination frequency along chromosome 4 was not evenly distributed, and the long arm had a much higher recombination frequency than the short arm (Figure 5). We used clone 4–4 and 4–5 as the boundary of euchromatin/heterochromatin of long arm and short arm. The recombination frequency along euchromatin of long arm was 27.6 cM/15.9 FL, about 3.5 times higher than that of short euchromatin region (5 cM∼7.5 cM/12.1 FL). Additionally, recombination was inhibited significantly at the pericentromeric heterochromatin region of chromosome 4 (Figure 5). The FISH results showed that this area defined by clone 4–4 and 4–5, spanned approximately 21.2% (7.9 FL/37.3 FL) of the cytological length of chromosome 4, but occupied only about 6.7∼8.3% (2.5∼3.1 cM/37.3 cM) of the genetic distance. The recombination suppression was also observed at the telomere region of chromosome 4. SSR03049 and SSR22514 markers with same genetic position (0 cM) from the end of LG-4 were mapped at 0.7 FL and 1.9 FL cytogenetically (Figure 5 and Table 3).

In addition, we observed obvious difference in recombination frequency between chromosomes 3 and 4. On average, the overall recombination frequency of chromosome 3 was ∼2.04 cM/µm, which was calculated by dividing the length of pachytene chromosome 3 (∼55.3 µm) by genetic length of LG-3 (∼112.7 cM). However, the chromosome 4 had only a lower recombination frequency of 0.86 cM/µm, which is the value of 37.3 cM/43.5 µm.

### 5. The Estimation of Unassembled Regions of Genome Draft Based on the High Resolution Pachytene FISH

End sequencing of fosmids above had been used for the assembling of scaffolds of cucumber genome [3]. Therefore, in cucumber genome draft database, the actual sequence position of these fosmids had been mapped. The quality of sequence assembly can be evaluated by comparing the cytological position of these fosmids on pachytene chromosome with that in draft sequence. The sequence positions of these fosmids and their FISH cytogenetic positions along chromosomes 3 and 4 were illustrated in Figures 4 and 5, respectively.

The results showed that the orientation of all fosmids along the pachytene chromosomes 3 and 4 were concordant with the sequence position in genome draft. In general, the relative distance of individual fosmid in draft genome sequence was similar to that in pachytene chromosome 3. However, in chromosome 4, the pericentromeric region spaced by fosmid 4–4 and 4–5 contained about 18.5% of this chromosome, which was only about 4% of the corresponding draft genome sequence. This indicated that substantial proportion of this region was missing in the draft genome sequence of chromosome 4. These results showed the genome draft of cucumber 9930 has a high assembly quality except for parts of heterochromatin, especially in chromosome 4.

### 6. The Distribution of Gene Density Along the Chromosomes 3 and 4

Cucumber 9930 has been sequenced, and the information of annotated genes along the chromosomes is available [3]. According to the Cucumber Genome Database (http://cucumber.genomics.org.cn/page/cucumber/index.jsp), the numbers of annotated genes along chromosomes 3 and 4 are ∼5260 and ∼3000, respectively. Both of them had ∼130 genes per Mb based on the draft.

**Figure 6:**
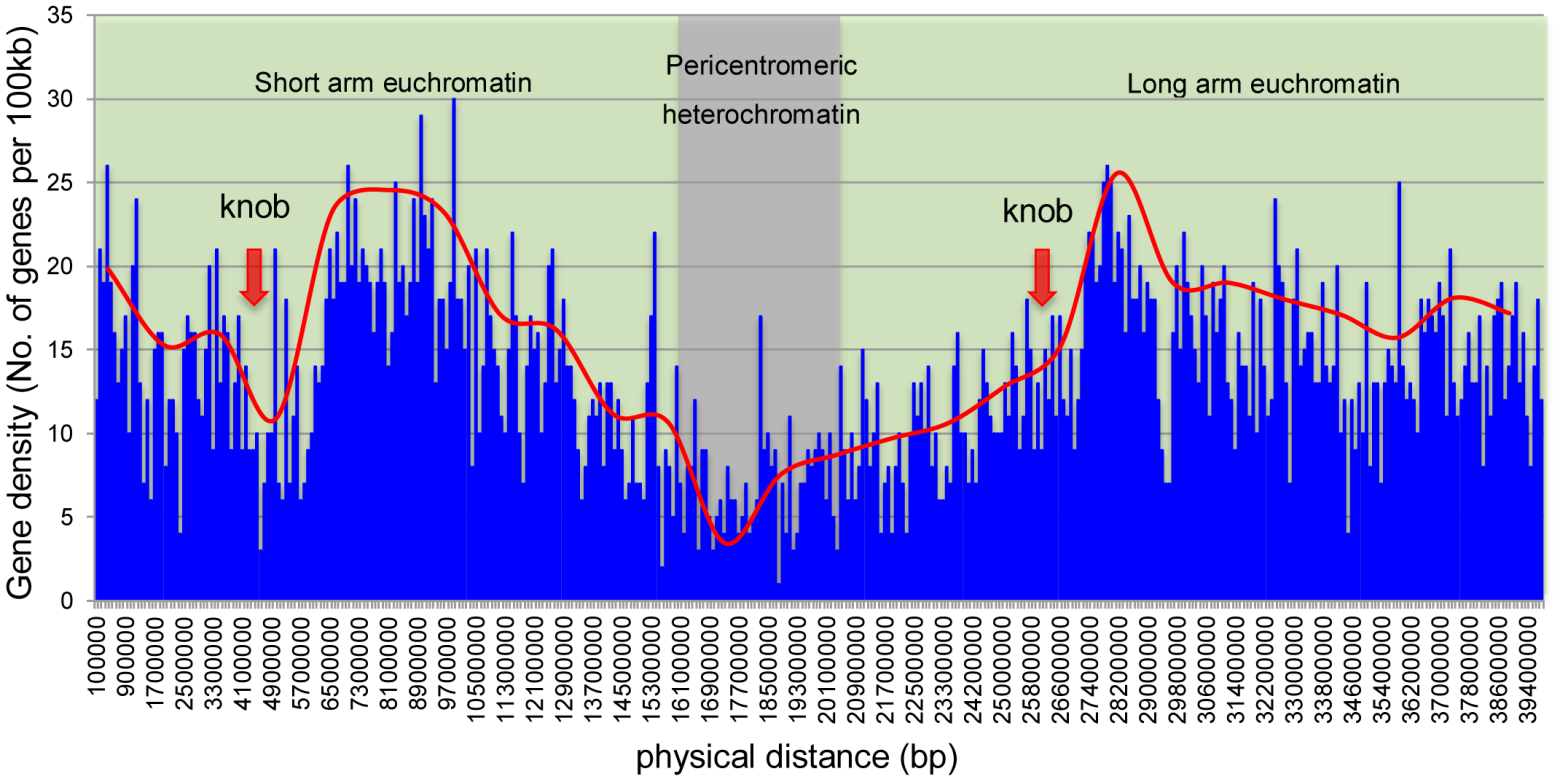
Distribution of gene density along the chromosome 3 of cucumber. The predicted gene location and number were from the cucumber genome draft published by Huang et al. *(2*009). They were accessed through Cucumber Genome Database (http://cucumber.genomics.org.cn/page/cucumber/index.jsp). The number of genes per 100 kb was plotted against the physical distance along the chromosome (in base pair). The red line showed the overall trend of gene density. The positions of knobs in the both arms were pointed by red arrows. Three regions, the short arm (green), the long arm (green), and the pericentromeric heterochromatin region (gray) were divided along the chromosome according to the structure of pachytene chromosome and FISH results.

**Figure 7:**
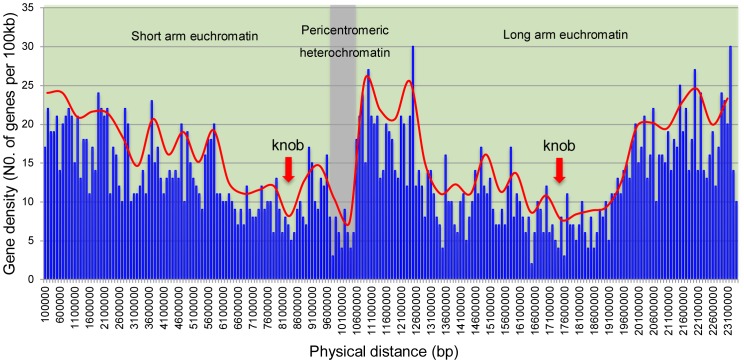
Distribution of gene density along the chromosome 4 of cucumber. The predicted gene location and number were from the cucumber genome draft published by Huang et al. (2009). They were accessed through Cucumber Genome Database (http://cucumber.genomics.org.cn/page/cucumber/index.jsp). The number of genes per 100 kb was plotted against the physical distance along the chromosome (in base pair). The red line showed the overall trend of gene density. The positions of knobs in the both arms were pointed by red arrows. Three regions, the short arm (green), the long arm (green), and the pericentromeric heterochromatin region (gray) were divided along the chromosome according to the structure of pachytene chromosome and FISH results.

The number of annotated genes per 100 kb was calculated to investigate the gene density along chromosomes 3 and 4. The distributions of gene density along both chromosomes were illustrated in Figures 6 and 7, respectively. Both of chromosomes were divided into three regions, short arm euchromatin (green color), long arm euchromatin (green color) and pericentromeric heterochromatin (gray color) regions based on the cytogenetic map and sequence information shown in Figures 4 and 5. In general, the uneven distribution of gene density was observed along both chromosomes 3 and 4. Both had the lowest gene density on the region of the centromere domains (Figures 6 and 7). However, patterns of gene distribution along chromosomes 3 and 4 were different.

For chromosome 3, the highest gene density regions appeared in the euchromatin domains of short arm and long arm. The lower gene density was observed towards the centromere and slightly decreased towards the ends of chromosome arms. Meanwhile, a significant decrease of gene density was observed on the knob domain of the short arm (Figure 6). For chromosome 4, overall, the lower gene density was observed towards the centromere and the higher gene density towards the telomeres. Relatively low gene density regions were also observed on the cytological knobs positions in the short arm and the long arm (Figure 7). Interestingly, a significantly high gene density region, ∼195 genes per Mb, was observed adjacent to the pericentromeric heterochromatin in the long arm, which ranged from ∼10.6 Mb to ∼12.5 Mb (Figure 7). In the integrated molecular cytogenetic map of chromosome 4, three fosmid clones, 4–5, 4–6, and 4–7 from this region showed significantly high recombination frequency than other regions (Figure 5).

## Discussion

The difference of recombination frequency along chromosome had been reported in many species [18], [20], [26]. The severe recombination repression has been frequently found on the pericentromeric heterochromatin domains in higher plants, which leads to gathering distribution of multiple molecular markers in closely spaced positions of linkage map [17], [18], [20], [27]. In the LG-4 of linkage map of cucumber [6], other 35 SSR markers were mapped on the same genetic position with marker SSR01601 which was cytogenetically positioned adjacent to heterochromatin in this research. The integrated molecular cytogenetic map revealed the significantly low recombination frequency on that region spaced by SSR01601 and SSR23826, which was defined as cytogenetically centromere and pericentromeric heterochromatin (Figure 5). This observation is different from the report by Yang et al. [8], in which no suppression of genetic recombination was observed. In fact they did not integrate the genetic map and chromosome map. Instead, they integrated the genetic and physical/sequence map of cucumber. It is believed from previous studies that due to the assembling difficult of tandem repeats, the physical/sequence map frequently lacks large blocks of tandem repeats [3], [28]. Therefore, the comparison between genetic map and physical/sequence map could not provide enough information. In addition, in the present research, the recombination suppression was also found at the end of chromosome 4, where SSR03049 and SSR22514 from the starting genetic positions (0 cM) were mapped onto discernible different cytogenetic position. In contrast to other higher plants, such as potato [18], papaya [20], rice [17], main satellites DNAs of cucumber, such as Type I/II and Type IV also exist in the chromosome ends, which might be the main cause for the recombination suppression in this area.

In this research, we mapped 23 SSR marker-anchored fosmid clones in pachytene spreads of chromosomes 3 and 4. The majority of them showed concordant orders in genetic map and cytogenetic map, except for three markers, SSR13274, SSR02244 and CSWGATT0 in chromosome 3. All these three markers were mapped cytogenetically near or on heterochromatic region (Figure 4). This disparity of genetic positions and cytogenetic positions might be due to skew segregation caused by the recombination repression in the heterochromation region. Based on the cytogenetic maps, the centromere of LG-3 was positioned in the SSR markers CSWGATT0 and SSR05572, and the centromeric region of LG-4 was mapped in the markers SSR01601 and SSR23826. Elucidating of the difference of recombination frequency along chromosome will provide important information for the isolating of agronomic traits genes based positional cloning.

The difference of recombination frequency might be attributed to the sequence composition. Obvious positive correlation between gene density and recombination rate has been revealed in crops, such as papaya and sorghum [20], [27]. In cucumber chromosome 4 of this research, a close correlation between the gene density and the recombination frequency was observed. The most severe recombination suppression was found in the pericentromeric heterochromatin region, which has the lowest gene density (Figures 5 and 7). However, in cucumber chromosome 3, the correlation between gene density and recombination frequency was not obvious. Generally, the recombination frequency was distributed evenly along the chromosome 3, and severe repression of recombination of large region was not observed, even in the pericentromeric region. This is likely to be explained by the patterns of heterochromatin distribution in chromosome 3.

Chromatin structure based on the DAPI staining showed that, except for several main knobs, no large blocks of pericentromeric heterochromatin was found in chromosome 3 (Figure 1). However, this result would not eliminate the possibility of recombination repression existing in the small region surrounding the knob. The wrong mapping of three SSR markers, SSR13274, SSR02244 and CSWGATT0 might be attributed to the skew segregation data because of recombination repression happening in small range. The sizes of recombination repression regions vary significantly in different plant species [18], [29], [30]. Our results showed that the significant difference of recombination repression exists also in individual chromosome of the same species. A much lower recombination repression in chromosome 3 (∼2.04 cM/µm of recombination frequency) than chromosome 4 (∼0.86 cM/µm of recombination frequency) might explain the extremely difference of genetic length of LG-3 (112.7 cM) and LG-4 (37.3 cM).

In higher plants, repetitive sequences consist of up to 95% of nuclear DNA [31]. Among these, satellites with high array tandem repeats are the most characteristic cytological markers in chromosomes, such as centromere, subtelomere and rDNA. Due to the large arrays of tandem repeats, the sequence assembly of the regions containing these satellites is extremely difficult. This explains why these regions exist as gap in most of sequenced genomes of higher plants. In cucumber, in addition to rDNA, four types of satellites have been identified, including Type I/II, Type III and Type IV [25]. FISH analysis revealed these satellites primarily located in heterochromatic centromere regions or subtelomeric regions [25]. The majority of the unassembled genome regions in cucumber are likely to be satellites sequences [3].

In our present research, the comparison between sequence map and FISH physical map revealed the difference of sequence assembly integrity of individual chromosome. Our results showed that chromosome 3 of cucumber has a relative high coverage of sequence (Figure 4). However, a huge gap was found in the region of pericentromeric heterochromatin of chromosome 4, across by fosmid 4–4 and fosmid 4–5 (Figure 5). The integration of cytogenetic and sequence map provides important information which could revise and facilitate sequence assembly, because the distribution of unassembled regions, or gaps could be displayed clearly. Yang et al. [8] found five mis-assembled scaffolds in cucumber 9930 draft genome and one in cucumber Gy14 draft genome, respectively, based on the FISH technology. Meanwhile, high-resolution pachytene chromosome spreads showed us the structural characters of unassembled regions, which would contribute to employing suitable assembling strategies to close the gaps and polish the genome sequence.
